# Supplementary material for: Resolving the full spectrum of human genome variation using Linked-Reads
Source: Genome Res. 2019 Apr;29(4):635–45. doi: 10.1101/gr.234443.118 (PMC6442396; doi:10.1101/gr.234443.118)
Supplement: Supplemental Material [file supp_gr.234443.118_Supplemental_longranger-2.2.zip › longranger-2.2.tar.bz2!/lr2.2-public/lib/stan/cmdstan/stan/doc/api/html/fwd_2functions_2sqrt_8hpp_source.html]

Stan: src/stan/agrad/fwd/functions/sqrt.hpp Source File


|  |  |
| --- | --- |
|  | Stan  2.5.0  probability, sampling & optimization |


- Main Page
- Related Pages
- Namespaces
- Classes
- Files

- File List
- File Members

All Classes Namespaces Files Functions Variables Typedefs Enumerations Enumerator Friends Macros Pages

- src
- stan
- agrad
- fwd
- functions

sqrt.hpp

Go to the documentation of this file.

1 #ifndef STAN\_\_AGRAD\_\_FWD\_\_FUNCTIONS\_\_SQRT\_HPP

2 #define STAN\_\_AGRAD\_\_FWD\_\_FUNCTIONS\_\_SQRT\_HPP

3

4 #include <stan/agrad/fwd/fvar.hpp>

5 #include <stan/meta/traits.hpp>

6 #include <stan/math/functions/inv\_sqrt.hpp>

7

8 namespace stan {

9

10  namespace agrad {

11

12  template <typename T>

13  inline

14  fvar<T>

15  sqrt(const fvar<T>& x) {

16  using std::sqrt;

17  using stan::math::inv\_sqrt;

18  return fvar<T>(sqrt(x.val\_), 0.5 \* x.d\_ \* inv\_sqrt(x.val\_));

19  }

20  }

21 }

22 #endif

stan::agrad::sqrt

var sqrt(const var &a)

Return the square root of the specified variable (cmath).

**Definition:** sqrt.hpp:51

stan::agrad::fvar

**Definition:** fvar.hpp:13

traits.hpp

stan::math::inv\_sqrt

boost::math::tools::promote\_args< T >::type inv\_sqrt(const T x)

**Definition:** inv\_sqrt.hpp:12

stan::agrad::sqrt

fvar< T > sqrt(const fvar< T > &x)

**Definition:** sqrt.hpp:15

fvar.hpp

inv\_sqrt.hpp

stan::agrad::fvar::d\_

T d\_

**Definition:** fvar.hpp:16

stan::agrad::inv\_sqrt

fvar< T > inv\_sqrt(const fvar< T > &x)

**Definition:** inv\_sqrt.hpp:15

stan::agrad::fvar::val\_

T val\_

**Definition:** fvar.hpp:15


---

[ Stan Home Page ]

*© 2011–2014,
Stan Development Team.*
